# The Second Law of Thermodynamics as a Force Law

**DOI:** 10.3390/e20040234

**Published:** 2018-03-28

**Authors:** Jürgen Schlitter

**Affiliations:** Biophysics, Ruhr-University Bochum, 44780 Bochum, Germany; juergen.schlitter@rub.de

**Keywords:** statistical mechanics, irreversibility, arrow of time, chemical dynamics

## Abstract

The second law of thermodynamics states the increase of entropy, ΔS>0, for real processes from state A to state B at constant energy from chemistry over biological life and engines to cosmic events. The connection of entropy to information, phase-space, and heat is helpful but does not immediately convince observers of the validity and basis of the second law. This gave grounds for finding a rigorous, but more easily acceptable reformulation. Here, we show using statistical mechanics that this principle is equivalent to a force law 〈〈f〉〉>0 in systems where mass centers and forces can be identified. The sign of this net force—the average mean force along a path from A to B—determines the direction of the process. The force law applies to a wide range of processes from machines to chemical reactions. The explanation of irreversibility by a driving force appears more plausible than the traditional formulation as it emphasizes the cause instead of the effect of motions.

## 1. Introduction

The second law of thermodynamics (SLT) is a fundamental and general empirical law of physics that is being discussed since it was articulated by Clausius [1] a long time ago. It states the permanent growth of entropy in the dynamics of all macroscopic systems from chemistry over biological life and engines to cosmic events and thus claims the existence of an arrow of time that seems to contradict the time-reversibility of classical and quantum mechanical theory. A second difficulty lies in the abstractness of the entropy concept. The connection between information and phase-space volume is still too far apart from typical real processes. The increase of that volume after the release of a constraint can be considered as self-evident [2]. Mathematically, it was in fact shown that the onset of motion after the release of a constraint follows the SLT [3], but this glance at a particular situation does hardly increase the plausibility for the fact that the increase of entropy represents the dynamical background of the everyday macroscopic world.

The SLT represents a universal dynamic law of macroscopic systems without explicitly referring to any force. Entropic forces, however, are known to be responsible for the isothermal pressure of an ideal gas, the elasticity of rubber [4] and macromolecular structure via internal fluctuations [5]. In these examples, entropy essentially provides static forces stabilizing equilibria. This also holds for entropic hydrophobic force in aqueous solutions, which, however, is also qualitatively considered as a cause of the protein folding process [6].

It was the aim of this study to find a clear, general and didactically accessible relationship between the change of entropy and a driving force that can be made responsible for the process. Such a relationship is not a theoretical proof of the second law, but it leads to an alternative formulation as a force law. The new look at the second law is also elucidating the occurrence of irreversibility.

The concept of a force is, of course, restricted to classical mechanics. Therefore, the following considerations apply from macroscopic processes down to molecular motions and reactions as long as they occur in the electronic ground state where, according to the Born–Oppenheimer approximation, nuclei effectively move on a potential energy surface. It is also assumed that classical statistical mechanics applies, which will be discussed later in the context of chemical reactions. The generalization to real quantum phenomena seems possible but is beyond the scope of this work.

## 2. General Approach

Let us consider a small system S characterized by only a few coordinates $x=\left(x_{1}\ldots x_{M}\right)$ like the position of pistons or similar moving parts, but also of interatomic distances involved in a chemical reaction. The composite system consists of a large number of N>>M particles where the environment has spatial and momentum coordinates $q=\left(q_{1}\ldots q_{3N-M}\right),p=\left(p_{1}\ldots p_{3N-M}\right)$ that are to be treated statistically. It is assumed that there is a Hamiltonian function

(1) H(q,p;x)=K(q,p)+v(q,p,x),

with $x=\left(x_{1}\ldots x_{M}\right)$ as parameters and a classical treatment is suited to treat equilibria and processes. K(q,p) is the kinetic and v(q,p,x) the potential energy.

**Figure 1:**
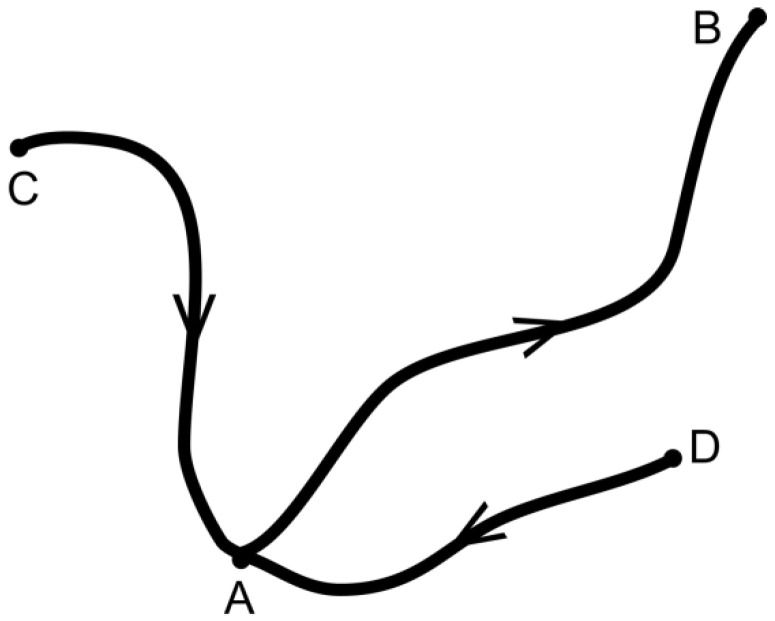
Pathways including state A. For possible processes, the arrows show the preferential direction that can be derived from the second law of thermodynamics or the new force law.

For the time being, we define states like A, B by representative configurations x, while a process is defined as a transition A→B, for instance. Figure 1 illustrates the existence of a few states near the starting state A and pathways connecting them.

By application of the Nabla operator $\nabla_{x}={\left(d/dx_{1}\ldots d/dx_{M}\right)}^{T}$, the Hamiltonian H(q,p;x) yields *M*-dimensional force vectors $F\left(q,p;x\right)=-\nabla_{x}H\left(q,p;x\right)$. The average behavior is determined by the potential of mean force (PMF) Π(x) via the mean force itself, ${\langle F\left(x\right)\rangle }_{\in }=-\nabla_{x}\Pi \left(x\right)$, which arises from integration over (q,p) in the statistical ensemble ∈, and examples will be given below. It vanishes at a stable thermodynamic state, which may be due to a constraint or occurs spontaneously and enables definition of thermodynamic potentials.

We now consider a process where the system follows a path from A to B, which is defined by x(l),0≤l≤L, $x_{A}=x(0)$ and $x_{B}=x(L)$ being representative positions. In physical chemistry, the use of a reaction coordinate l is a common means of parameterizing reaction pathways. x(l) describes a one-dimensional path in the *M*-dimensional space of the system S like, for instance, the position of an atom on its way from one binding partner to the next in the three-dimensional space. The definition allows simultaneous and subsequent motions of mass centers. The direction is given by increasing *l*, and *L* is the length of the path as |dx/dl|=1. The component of the force in the direction of the path is f(l)=F(q,p;x)·dx/dl. The average along the path of the corresponding mean force ${\langle f(l)\rangle }_{\in }$ becomes

(2) $${\langle {\langle f\rangle }_{\in }\rangle }_{AB}=L^{-1}\int_{0}^{L}{\langle F\left(x\right)\rangle }_{\in }\cdot \frac{dx}{dl}dl=-L^{-1}\left(\Pi \left(x_{B}\right)-\Pi \left(x_{A}\right)\right)\equiv -\Delta \Pi_{AB}/L.$$

Note that the dot product here denotes the *M*-dimensional inner product. Interestingly, the average mean force is independent of the pathway itself and depends only on the difference of the PMF at the endpoints. The sign indicates the direction of the process as ${\langle {\langle f\rangle }_{\in }\rangle }_{BA}=-{\langle {\langle f\rangle }_{\in }\rangle }_{AB}$. The crucial result is the equivalency

(3) $${\langle {\langle f\rangle }_{\in }\rangle }_{AB}>0\Leftrightarrow \Delta \Pi_{AB}<0.$$

This is the very general form of the force law, which, for the sake of clarity, will be illustrated by considering two important ensembles.

The computation of PMFs from the mean force is an established procedure for obtaining free energy differences with many applications in chemistry and biology [7,8,9]. Often isobaric-isothermal conditions are assumed, which we also consider below. So far, however, no particular attention was given to the relationship Equation (3) between the signs of the mean force and the PMF, which is in the focus of this article.

## 3. Results

### 3.1. Microcanonical Ensemble: The Force Law and the Increase of Entropy

For an NVE ensemble, entropy is determined by the phase-space volume of a shell of width ΔE between the energy surfaces H=E and H=E+ΔE. Here, we consider systems that are large enough (sufficiently large particle number *N*) to apply the thermodynamic limit where entropy becomes independent of ΔE and can be written [10]

(4) $$S(x)=k_{B}\ln\int_{V}d\Gamma \Theta \left(E-H\left(q,p;x\right)\right).$$

Integration is restricted to the given volume V. The differential phase volume is $d\Gamma =dq^{3N-M}dp^{3N-M}/h^{3N-M}$ for non-identical particles and has to be adapted for other cases, h the Planck constant and $k_{B}$ the Boltzmann constant. At chemical reactions, it is possible to deal with constant particle numbers $n_{i}$ and $N=\sum n_{i}$ when not molecules, but nuclei are treated as different kinds of particles.

Θ(y) is the Heaviside jump function that equals 1 at y>0 and zero elsewhere. Its derivative is the functional δ(y), which is zero for y≠0. It enters the probability density

(5) $$p=\delta \left(E-H\left(q,p;x\right)\right)Q^{-1};\;Q=\int_{V}d\Gamma \delta \left(E-H\left(q,p;x\right)\right)$$

that vanishes wherever H≠E. When entropy is differentiated with respect to x, one obtains

(6) $$\begin{array}{cc} \nabla_{x}S(x) & =-k_{B}\int_{V}d\Gamma \delta \left(E-H\left(q,p;x\right)\right)\nabla_{x}H\left(q,p;x\right)\;Q^{-1}/\int_{V}d\Gamma \Theta \left(E-H\left(q,p;x\right)\right)\;Q^{-1}\\ & ={\langle F(x)\rangle }_{NEV}/{\left(\partial S/\partial E\right)}^{-1}\\ & ={\langle F(x)\rangle }_{NEV}/T(x) \end{array}$$

Note that $Q^{-1}$ was inserted twice to produce interpretable expressions in the numerator and denominator. The positive quantity $T={\left(dS/dE\right)}^{-1}$ is the temperature of the complete system and can, therefore, be considered as constant in the thermodynamic limit of large total systems, i.e., for N>>M. Then, the mean force is ${\langle F(x)\rangle }_{NEV}=T\nabla_{x}S(x)$ and the PMF becomes Π(x)=−TS(x). Insertion of the PMF in Equation (2) finally yields the crucial statement according to Equation (3)

(7) $${\langle {\langle f\rangle }_{NVE}\rangle }_{AB}>0\Leftrightarrow \Delta S_{AB}>0.$$

The claim of the SLT for a real process is an increase of entropy, $\Delta_{AB}S>0$. This is apparently equivalent to the force law for a real process, ${\langle {\langle f\rangle }_{NVE}\rangle }_{AB}>0$, which claims a positive net force, i.e., average mean force along any pathway A→B.

It is easily seen that the force law Equation (7) holds even at processes in small environments where temperature does not remain constant when the average Equation (2) along the path is taken with weights proportional to 1/T(x).

### 3.2. Isobaric-Isothermal Ensemble: The Force Law and the Decrease of Gibbs Energy

In life science and chemistry, the NPT ensemble applies to the majority of cases where temperature *T* and pressure *P* are maintained by a loosely coupled large environment. Here, one starts from Gibbs energy [8], which can be expressed by the configurational integral as

(8) $$G(x)=-k_{B}T\ln\left(\beta P\int d\Gamma \int dV\exp\left(-\beta \left(H\left(q,p;x\right)+PV\right)\right)\right)$$

with $\beta =1/k_{B}T$ [11]. The *N*–particle system comprises at least those atoms that are strongly interacting with the system S by, for instance, covalent bonds, but the assumption N>>M can be dropped. The probability density is proportional to exp(−β(H(q,p;x)+PV)) and yields the ensemble mean of the force ${\langle F(x)\rangle }_{NPT}=-\nabla_{x}G(x)$ when Equation (6) is differentiated. Therefore, the PMF equals the Gibbs energy Π(x)=G(x), and it follows from Equation (3) that a real process is characterized by two equivalent assertions

(9) $${\langle {\langle f\rangle }_{NPT}\rangle }_{AB}>0\Leftrightarrow \Delta_{AB}G<0.$$

Here, the force law is equivalent to the well-known claim for Gibbs energy that it will decrease at a real process, which is a consequence of the SLT for the NVE ensemble.

Interestingly, the mean force can be measured either experimentally in the case of macroscopic systems like engines, or computationally for microscopic systems like protein nanomachines or even atoms at a chemical reaction. The principle is like this: the system is stopped at some position x and the force required for that purpose, and the so-called mean constraint force, $F_{c}$, is measured, which coincides with the negative mean force as [8,12,13]

(10) $${\langle F\left(x\right)\rangle }_{\in }=-{\langle F_{c}\left(x\right)\rangle }_{\in }$$

at Cartesian or distance coordinates [14]. The constraint force usually exhibits strong fluctuations and convergence towards the mean that depends on relaxation processes of the environment.

### 3.3. Concluding Remarks

**Figure 2:**
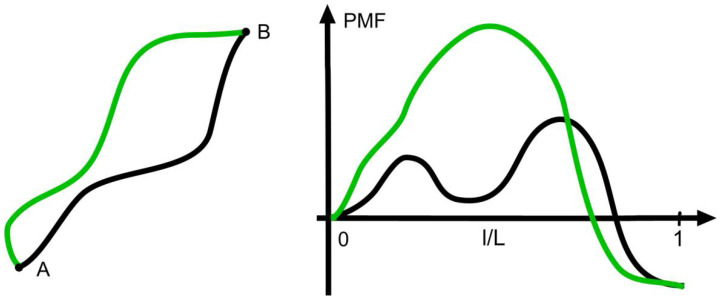
Alternative pathways from A to B (left) with respective profiles of the potential of mean force. In either case, the identical decrease of the potential of mean force (PMF) implies that the average mean force—i.e., the negative derivative—along the path is positive and drives the motion from A to B. Likewise, the positive average mean force implies the decrease of the PMF.

Given the connection between the signs of the net force ${\langle {\langle f\rangle }_{\in }\rangle }_{AB}$ and the change in entropy, one could assume that the strength of the net force is also connected with the velocity or rate of the transition considered. This is not the case as can be seen from Figure 2, which shows two different pathways with the same decrease of the PMF, which, according to Equation (2), is

(11) $$\Delta \Pi_{AB}=-{\langle {\langle f\rangle }_{\in }\rangle }_{AB}L.$$

According to theories of activated processes, the rate of a transition along a given path is determined by the maximum of the PMF on the way A→B [15], and, therefore, the pathways shown are taken with different rates despite the same $\Delta \Pi_{AB}$. On the other hand, pathways with the same maximum can have different values of ${\langle {\langle f\rangle }_{\in }\rangle }_{AB}L$ at an unchanged transition rate.

The net force determines, however, the equilibrium between two states, which can be expressed by ratio $k_{AB}/k_{BA}$ of the rates $k_{AB}$ for process A→B and $k_{BA}$ for the reverse process B→A. Under NPT conditions and following theories of activated processes [15], the ratio is connected with the change of Gibbs energy $\Delta G_{AB}=\Delta \Pi_{AB}$. Using Equation (11), one obtains

(12) $$\frac{k_{AB}}{k_{BA}}=\exp\left(-\Delta G_{AB}/k_{B}T\right)=\exp\left({\langle {\langle f\rangle }_{\in }\rangle }_{AB}L/k_{B}T\right).$$

At negative $\Delta \Pi_{AB}=\Delta G_{AB}$ like in Figure 2, the forward process occurs at a higher rate than the reverse process as the net force favors the forward process and opposes the other direction. In the world of molecular processes like chemical reactions, one occasionally finds considerable rates of back-reactions. At macroscopic transitions, however, they are extremely rare with minute values of $k_{BA}/k_{AB}$, which, according to Equation (12), are due to forces and distances covered that are far above molecular orders of magnitude.

## 4. Discussion

We have addressed the SLT using the concept of pathways and their characterization by mean forces depending on the ensemble chosen. As a result, we propose a new force law stating that a real thermodynamic process is based on a net force with the right sign. This alternative and equivalent formulation of the SLT holds for processes from machines down to chemical reactions as long as they can be parametrized by motions of a few mass centers of a much larger system. Heat conduction itself and photochemical reactions are not included as forces do not enable a reasonable formulation of such processes.

The application to chemical reactions including processes of biological life like enzyme catalysis, where bonds are broken and others are formed, deserves particular consideration. For a given Born–Oppenheimer potential energy function (BOP), quantum mechanics still requires consideration of tunneling and transmission coefficients when calculating transition rates [16], zero-point energies and entropies when calculating equilibrium quantities as we do here. Without quantum mechanical correction, BOP would yield negative entropy [17] and too low energy for chemical bonds and is only an approximation to the classical force field v(q,p,x) that produces correct Gibbs bond energies. This force field will differ from BOP by shallower potential wells for chemical bonds (and bond angles) and slightly depends on temperature. Considerable efforts are still made to construct such force fields [18]. For the force law discussed here, it only matters that quantum mechanics suggest the existence of a classical force field that yields the correct PMF in Equation (2).

Newton’s second law connects force with acceleration by a rigorous analytic relation independent of the direction of the motion. It is time-reversible since it holds in either direction and does not imply that motion follows the force. The new force law 〈〈f〉〉>0, however, which is equivalent to the SLT ΔS>0 in the world of mass centers, is a statistical statement that reflects the behavior of the underlying ensemble. Given two states A and B, it gives preference to a particular direction, the other direction being drastically suppressed in macroscopic circumstances. On the average, motion follows the mean force. Apparently, the arrow of time is represented by the arrow of the net force originating from a realistic ensemble. The deeper reason for irreversibility is, of course, the occurrence of relaxation, which is always tacitly assumed when using ensembles and mean values.

The above derivations of the force law are based on the correct probability distributions via their partition functions, i.e., the integrals occurring in Equations (4) and (6). The importance of this prerequisite is demonstrated by the counterexample of a time-reversible system, the undamped pendulum. Taking the time average, one recognizes that the pendulum is preferably at high potential energy. Only if damping is introduced by coupling to a heat bath, both irreversible behavior and a realistic distribution do emerge, which confirms the close connection between the two features.

Despite the mathematical equivalency, there is a clear difference between the traditional form of the SLT and the force law. While the SLT talks about entropy as the characteristic product of a real process, the force law emphasizes the cause of real processes by stating that a net driving force is needed and determines the direction. It offers the didactical advantage of clarifying much of the SLT on the familiar basis of forces before introducing entropy as a concept of statistics or information theory. One may hope that the SLT as a force law is a more plausible and easily acceptable explanation of reality.
